# Supplementary material for: Effects of Ferric Ions on Cellulose Nanocrystalline-Based Chiral Nematic Film and Its Applications
Source: Polymers (Basel). 2024 Jan 31;16(3):399. doi: 10.3390/polym16030399 (PMC10856810; doi:10.3390/polym16030399)
Supplement: Supplementary file 1 [file polymers-16-00399-s001.zip › polymers-2846201-supplementary.pdf]

Effects of Ferric Ions on Cellulose Nanocrystalline-Based Chiral Nematic Film and Its Applications

Shuaiqi Wang, Bingqun Lin, Yihan Zeng and Mingzhu Pan *

List of Supplementary Information

2.2 Preparation of CNC/$FeCl_3$ colloids

15 g of MCC was hydrolyzed for 50 min for preparation of the suspension. Hydrolysis was performed with 64 wt. % sulfuric acid at acid-to-pulp ratio of 8:1 at 45 °C and 60 °C. The suspension was then diluted 10-fold to stop the reaction, and then further diluted with the deionized water and centrifuged for three cycles. The sample was placed inside dialysis membrane tubes and dialyzed against slow-running deionized water for 2 to 4 days until the pH of supernatant was neutral. Subsequently, the colloidal solution was dispersed by ultrasound treatment in an ultrasonic cell crusher (X-1200D, ATPIO, China) for 25 min. Finally, CNC suspension was condensed using a rotary evaporator (RE-501A, ATPIO, China) until the concentration of CNC reached 2.0 wt.%.

2.3 Characterizations

The morphology of CNC and CNC/$FeCl_3$ chiral nematic films were investigated with transmission electron microscope (TEM, JEM-1400, JEOL, Japan), and field-emission scanning electron microscope (FE-SEM, HITACHI S4800, Thermo Scientific, USA). The chiral nematic films were also conducted by atomic force microscope (AFM, Dimesion Edge, Bruker, Germany) in a tapping mode using the commercial silicon probe with tip radius of 8 nm and microcantilever length of 125 μm at a scanning speed of 1.0 Hz, scanning angle of 0°, and tapping frequency range of

340~380 kHz, respectively. chiral nematic liquid crystal behavior during ESIA was measurement with polarization optical microscope (POM, BX41, Olympus, Japan).

The structure of CNC and CNC/$FeCl_3$ chiral nematic films were investigated with Fourier-infrared spectra (FTIR, VERTEX 80, Bruker, Germany), UV-Vis spectra (Lambda 950, PE, USA), circular dichroism (CD) spectra (J-1500, Jasco, Japan), X-ray diffraction (XRD, Ultima IV, Rigaku, Japan). The crystallinity index (*CrI)* was calculated according to Equation (1) [27]:

$$CrI(\%) = \frac{A_{total} - A_{am}}{A_{total}} * 100 \qquad (1)$$

Where A_{total} and A_{am} represent the total area of the diffract gram, and the area of the amorphous peak, respectively.

The zeta potential values of CNC/$FeCl_3$ colloid were measured by a nano zetasizer (Zetasizer Nano ZS, Malvern, UK) based on the principles of dynamic light scatting.
